# The seventh ENBDC workshop on methods in mammary gland development and cancer

**DOI:** 10.1186/s13058-015-0629-5

**Published:** 2015-09-02

**Authors:** Marina A. Glukhova, Nancy Hynes, Maria dM Vivanco, Renée van Amerongen, Robert B. Clarke, Mohamed Bentires-Alj

**Affiliations:** Institut Curie, PSL Research University, 26 Rue d’Ulm, Paris, F-75248 France; CNRS, UMR144, 26 rue d’Ulm, Paris, F-75248 France; INSERM, 101 rue de Tolbiac, Paris, F-75013 France; Friedrich Miescher Institute for Biomedical Research (FMI), Maulbeerstr. 66, CH-4058 Basel, Switzerland; CIC bioGUNE, Cell Biology & Stem Cell Unit, Technological Park of Bizkaia, 801 A, 48160 Dero, Bizkaia, Spain; Section of Molecular Cytology and Van Leeuwenhoek Center for Advance Microscopy, Swammerdam Institute for Life Sciences, University of Amsterdam, Science Park 904, 1098 XH Amsterdam, The Netherlands; Institute of Cancer Sciences, Paterson Building, University of Manchester, Wilmslow Road, Manchester, M20 4BX UK

## Abstract

The seventh annual meeting of the European Network of Breast Development and Cancer Laboratories, held in Weggis, Switzerland, in April 2015, was focused on techniques for the study of normal and cancer stem cells, cell fate decisions, cancer initiation and progression.

## Introduction and keynote

This year, once more, at the foot of the Rigi Mountain, by the lake of Lucerne, the idyllic resort town of Weggis, admired by Mark Twain and Sergei Rachmaninoff, welcomed the annual meeting of the European Network of Breast Development and Cancer Laboratories. The keynote lecture delivered by Christine Watson (Cambridge University, UK) set the scientific tone of the meeting at a high level. Watson talked about transcriptional control of cell fate decisions in the mammary gland and, in particular, about the role played by the signal transducer and activator of transcription (STAT) family of transcription factors. A novel regulator of mammary cell proliferation, the transcription factor Roma, a KRAB domain Zinc Finger protein, is regulated by STAT6. Roma overexpression in mammary epithelium induces an upregulation of p21 and a G_1_ arrest, whereas Roma deletion leads to low p21 levels, an increase of luminal cell proliferation, and accelerated alveologenesis. In the second part of her talk, Watson showed that, despite general belief to the contrary, programmed cell death in the involuting gland is mediated by lysosomal cathepsins rather than caspases [1]. STAT3 regulates the size of the lysosomal compartment, the expression of cathepsin inhibitor Spi2A, and the uptake of milk fat globules. Milk fat induces leakage of cathepsins from lysosomes, leading to cell death [2].

## Session 1: Cell hierarchy in human breast and breast cancer (chairs: Maria dM Vivanco and Renée van Amerongen)

The first session focused on the epithelial cell hierarchy in breast cancer. Daniel Birnbaum (Centre de Recherche en Cancérologie de Marseille, France) discussed the identification and targeting of breast cancer stem cells (CSCs). His group used Aldefluor positivity as a marker for CSCs in basal and ERBB2^+^ breast cancers, allowing them to identify specific DNA methylation profiles in the CSC population [3]. The investigators also discovered that histone desacetylase inhibitors induced breast CSC differentiation but only in tumors with low expression of long non-coding RNA *XIST*, suggesting the potential of *XIST* as a predictive biomarker [4].

Mohamed Bentires-Alj (Friedrich Miescher Institute for Biomedical Research, Basel, Switzerland) discussed the clinical implications of tumor heterogeneity. He highlighted the tumor-initiating and tumor-promoting effects of the tyrosine phosphatase SHP2 [5, 6] and showed data demonstrating that phosphatidyl-inositol-3-kinase mutation can overcome lineage restriction, resulting in cell fate switching during mammary tumor formation. In the second part of his talk, Bentires-Alj showed that one can interfere with the tumor microenvironment to bypass the issue of tumor heterogeneity. The neutralization of the chemokine (C-C motif) ligand 2 (CCL2) in mice inhibits metastasis by retaining monocytes in bone marrow, but cessation of anti-CCL2 treatment triggers an overshoot of metastases in preclinical models of metastatic breast cancer [7]. Bentires-Alj emphasized the need for long-term follow-up of patients with metastatic disease after anti-CCL2 treatment.

Richard Iggo (Institut Bergonie, Bordeaux, France) talked about his work to humanize the luminal layer of the mouse mammary gland by intraductal injection of normal breast cells transduced with five oncogenes. This approach may facilitate the study of defined steps during human mammary epithelial cell tumorigenesis by providing a more physiological environment than previous methods [8]. Furthermore, Iggo discussed his lab’s efforts to test drugs by comparing the response to targeted therapy of genetically defined human tumor xenografts in mice.

The session concluded with a short talk by Sven Rottenberg (Institute of Animal Pathology, Bern, Switzerland), who discussed the efficacy of poly(ADP-ribose) polymerase (PARP) inhibitors such as olaparib in homologous recombination-deficient *BRCA1*-null tumors. Unfortunately, homologous recombination is frequently restored in tumors that acquire PARP inhibitor resistance, and Rottenberg described his hunt for the responsible molecular mechanisms, revealing *Rev7* loss as the latest culprit [9].

## Session 2: Pathways and mechanisms essential for mammary development and breast cancer (chairs: Robert Clarke and Nancy Hynes)

Keith Brennan (University of Manchester, UK) presented a new model of Notch-dependent mammary tumors, the MMTV-VP16/RBPj mouse strain. The transcription factor RBPj normally requires Notch signaling for its activation. MMTV-VP16/RBPj mice express a constitutively active RBPj form in the mammary gland in which RBPj is fused to the VP16 transcriptional activation domain. These transgenic mice display an expanded luminal compartment, but the ratio of estrogen receptor-positive (ER^+^) to ER-negative cells is not altered and the transgene is not expressed in ER^+^ luminal cells. Proliferation rates are decreased in the ducts of these mice but are elevated in the terminal end buds, and there are more cell divisions perpendicular to the basement membrane than in control mice. The post-weaning involution is delayed in transgenic animals, and, in vitro, VP16-RBPj-expressing cells are resistant to anoikis. Multiparous MMTV-VP16/RBPj mice develop ER^+^ invasive tumors at 10–15 months and associated lesions that resemble ductal carcinomas in situ. Within the tumors, VP16/RBPj-expressing cells are resistant to doxorubicin, whereas those negative for the fusion protein respond to the drug. Brennan discussed the possibility of using these transgenic mice as a model of endocrine-resistant ER^+^ human breast cancers.

Daniela Taverna (University of Turin, Italy) presented on miRNAs in breast cancer progression with a focus on miR-148b and miR-214 [10, 11]. miR148b expression in breast cancer cells impairs lung colonization in mouse models, suggesting a role in regulating genes contributing to metastasis. In vitro, miR148b expression in breast cancer cells increases cell adhesion but reduces cell invasion. Predicted RNA targets include *ITGA5*, *ROCK1*, *NRAS*, *CSF1*, and *ALCAM*. Experimental knockdown of *ITGA5* or *ALCAM* can reduce metastasis. In contrast, miR214 expression can lead to an increase in ALCAM, which suggests interactions with miR148b, perhaps suppressing its activity to coordinate metastatic dissemination. In breast cancer, there is evidence that miR214 and miR199 repress *FOXP2*, which in turn inhibits stem cell genes. Anti-miR214 therapies are currently in development in the laboratory.

Eva Gonzalez-Suarez (IDIBELL, Barcelona, Spain) talked about receptor activator of nuclear factor-kappa-B (RANK) signaling in breast cancer. Transgenic mice expressing RANK in the mammary epithelium have increased stem cell and luminal progenitor compartments, expanded luminal and basal cell populations, reduced expression of Elf5, the transcription factor required for lactogenic differentiation, and spontaneously develop mammary tumors [12]. Forced expression of the *RANK* gene in MCF10A normal immortalized breast cells induces epithelial-to-mesenchymal transition and stemness: acquisition of CD44^+^/CD24^−^ phenotype and the ability to grow in vivo [13]. RANK overexpression in breast cancer cell lines deficient for *BRCA1* increases tumorigenesis and metastasis. RANK is expressed in 50 % of the hormone receptor-negative human breast adenocarcinomas but in only 15 % of luminal breast cancers, and high RANK expression is associated with poor survival. In contrast to RANK, its ligand, RANKL, is rarely expressed in human breast cancer cells but is produced by tumor-infiltrating lymphocytes [13]. Thus, this stromal component of tumors could be a source of RANKL that signals to the tumor cells.

## Session 3: In vivo and in vitro analyses of cell behavior in development and cancer (chair: Mohamed Bentires-Alj)

Danijela Matic Vignjevic (Institut Curie, Paris, France) talked about “Mechanisms of epithelial cell migration: role of microenvironment” in gut homeostasis. Using intestinal slice cultures and intravital two-photon microscopy, Vignjevic’s team assesses whether epithelial cells migrate actively (using cellular protrusions) or passively (by being pushed by dividing cells) or are transported by underlying fibroblasts or basement membrane flow or both. This laboratory also studies the role of the microenvironment in cancer cell invasion. They use a native basement membrane to determine which cell type—cancer cells or fibroblasts—is degrading basement membrane and whether distinct subpopulations of fibroblasts have different effects. Using three-dimensional (3D) in vitro models, Vignjevic and colleagues investigate how fibroblasts assist cancer cell migration through the stroma: is it via stimulation of the invasive capacity of cancer cells or via generation of a pro-invasive extracellular matrix? Finally, using microfluidic 3D chemotaxis assay, they assess the mechanism of collective chemotaxis and whether fibroblasts serve as guides for cancer cells and help them to reach the blood vessels.

Matt Smalley (University of Cardiff, UK) talked about cleared fat pad transplantation (CFPT), a signature technique of the field [14]. Smalley started by describing CFPT and underlined the importance of the pioneering studies involving CFPT experiments [15] (and http://www.enbdc.org/conversations/gil_smith.html). However, given that CFPT is non-physiological, that flow cytometry cell sorting stresses the cells, and that the mammary epithelium is highly plastic, Smalley suggested that CFPT is more akin to a local metastasis assay. Moreover, lineage tracing and imaging tools to assess stem cell activity in situ were recently developed, and CFPT may no longer be the gold standard stem cell assay [16, 17]. Nonetheless, Smalley argues that CFPT will continue to be very useful for the mammary gland field: for addressing local versus systemic and paracrine effects (e.g., [18]), for identifying the functions of genes for which the knockout is embryonic lethal (e.g., [19]), and for in vivo functional developmental and tumor analyses. Indeed, CFPT remains important for estimating regenerative potential of cell populations (e.g., [20]), whereas serial transplantation may allow estimates of self-renewal potential.

## Conclusions

The quality of work presented during the sessions and high motivation of students and postdocs to communicate their latest findings during two poster sessions were greatly appreciated by meeting attendees. Owing to the limited number of participants, the meeting provided the possibility of easily exchanging methods and reagents, discussing technical pitfalls, and identifying laboratories for postdoctoral studies. The 8th European Network of Breast Development and Cancer Laboratories meeting will be chaired by Renée van Amerongen and will take place in Weggis on 12–14 May 2016. Following the suggestion of the meeting attendees, one of the sessions next year will be organized and chaired by a postdoc, Bethan Lloyd-Lewis (Cambridge University), and a Ph.D. student, Anoeska van de Moosdijk (University of Amsterdam).

## Abbreviations

3D: Three-dimensional
CCL2: Chemokine (C-C motif) ligand 2
CFPT: Cleared fat pad transplantation
CSC: Cancer stem cell
ER: Estrogen receptor
PARP: Poly(ADP-ribose) polymerase
RANK: Receptor activator of nuclear factor-kappa-B
RANKL: Receptor activator of nuclear factor-kappa-B ligand
STAT: Signal transducer and activator of transcription
